# Measuring Genome Sizes Using Read-Depth, k-mers, and Flow Cytometry: Methodological Comparisons in Beetles (Coleoptera)

**DOI:** 10.1534/g3.120.401028

**Published:** 2020-06-29

**Authors:** James M. Pflug, Valerie Renee Holmes, Crystal Burrus, J. Spencer Johnston, David R. Maddison

**Affiliations:** * Department of Integrative Biology, Oregon State University, Corvallis, OR 97331; † Department of Entomology, Texas A&M University, College Station, TX 77843

**Keywords:** Genome Size, K-mer, Carabidae, Flow Cytometry, Insect Genomes

## Abstract

Measuring genome size across different species can yield important insights into evolution of the genome and allow for more informed decisions when designing next-generation genomic sequencing projects. New techniques for estimating genome size using shallow genomic sequence data have emerged which have the potential to augment our knowledge of genome sizes, yet these methods have only been used in a limited number of empirical studies. In this project, we compare estimation methods using next-generation sequencing (k-mer methods and average read depth of single-copy genes) to measurements from flow cytometry, a standard method for genome size measures, using ground beetles (Carabidae) and other members of the beetle suborder Adephaga as our test system. We also present a new protocol for using read-depth of single-copy genes to estimate genome size. Additionally, we report flow cytometry measurements for five previously unmeasured carabid species, as well as 21 new draft genomes and six new draft transcriptomes across eight species of adephagan beetles. No single sequence-based method performed well on all species, and all tended to underestimate the genome sizes, although only slightly in most samples. For one species, *Bembidion* sp. nr. *transversale*, most sequence-based methods yielded estimates half the size suggested by flow cytometry.

The advent of modern genomic methods and the resulting deluge of data from next generation sequencing (NGS) has been a tremendous boon to genomic studies. In spite of this, many foundational questions about genomes have remained largely unanswered. One such question is why genomes vary so much in size: there is an over 3,000-fold difference between the smallest and largest genomes in animals (Gregory 2001). Revealing the myriad evolutionary causes behind this variation has proven to be a difficult and enduring challenge (Cavalier-Smith 1978, Elliott and Gregory 2015). One limitation to understanding genome size evolution is the relative lack of knowledge of genome sizes in some of the larger clades of life, such as the arthropods (Hanrahan and Johnston 2011).

Knowledge of genome size is desirable for multiple reasons. It can be important to understand phenomena such as whole genome duplication and polyploidy (Allen 1983, Némorin *et al.* 2013), genome reduction driven by changes in selective pressure (Johnston *et al.* 2004), or proliferation of non-coding DNA sequence (Gregory 2005). Genome size has been observed to correlate with a variety of developmental factors, such as egg size (Schmidt-Ott *et al.* 2009) and cell division rate (Gregory 2001). Knowledge about genome size can also be valuable in species delimitation. Differences sufficient to reproductively isolate a population into separate species may be difficult to distinguish using traditional morphological or DNA sequence data; however, such differences may be more apparent once genome sizes are taken into consideration alongside other evidence (Gregory 2005, Leong-Škorničková *et al.* 2007).

Traditional methods for determining genome size, such as Feulgen densitometry, Feulgen image analysis densitometry, and flow cytometry, are well-tested and generally reliable (Chen *et al.* 2015, Hanrahan and Johnston 2011, Hardie *et al.* 2002). However, these techniques rely on live, appropriately fixed, or frozen tissues with largely intact cells, effectively limiting study to organisms that can be raised in the lab or easily found in nature and transported to the lab (Hanrahan & Johnston 2011). This can be a problem if properly prepared material cannot be easily obtained, such as with extinct species, or those found in remote or inaccessible habitats. In these cases, dried or fluid-preserved museum specimens that are unsuitable for flow cytometry or Feulgen staining may be much more readily available. NGS can potentially provide a relatively simple alternative for bioinformatically estimating genome size; however, the accuracy and feasibility of these methods has not been extensively studied.

The first of the sequence-based methods we investigate uses k-mer distributions. K-mers are unique subsequences of a particular length, k, from a larger DNA sequence. For example, the DNA sequence AACCTG can be decomposed into four unique k-mers that are three bases long (referred to as 3-mers): AAC, ACC, CCT, and CTG. Any set of DNA sequences, including unassembled short reads produced by NGS, can be broken down into its constituent k-mers. Each unique k-mer can be assigned a value for coverage based on the number of times it occurs in a sequence (*e.g.*, if the 3-mer CTG is found a total of 20 times, it would have a coverage of 20). The distribution of coverages for all k-mers from a sequence can be plotted to produce a k-mer frequency distribution. For k-mers generated from genomic sequencing reads with negligible levels of sequence artifacts (sequencing errors, repeats, or coverage bias), the distribution of k-mer frequencies will approximate a Poisson distribution, with the peak centered on the average sequencing depth for the genome (Li & Waterman 2003). The value of k varies among analyses, though values ranging between 17 and 35 are typical (Chen *et al.* 2015, Liu *et al.* 2013).

Techniques for estimating genome size using k-mer distributions generally work best when the average coverage is greater than 10X (Williams *et al.* 2013), but newer methods with more comprehensive models for addressing sequencing errors and repetitive sequences are showing promise at coverages as low as 0.5X (Hozza, Vinař, & Brejová 2015). Examples of accurate k-mer based genome size estimates exist for a variety of organisms, including giant pandas (Li *et al.* 2010), cultivated potatoes (Potato Genome Sequencing Consortium 2011), the agricultural pest *Bemisia tabaci* (Chen *et al.* 2015), and oyster (Zhang *et al.* 2012). However, these methods can also produce ambiguous or incorrect estimates. K-mer analysis of genomic reads from a male milkweed bug produced estimates that were 60Mb to 1110Mb higher than the approximately 930Mb flow cytometry genome estimate (Panfilio *et al.* 2019), with the magnitude of this overestimation increased at larger values of k. A separate study on the *Bemisia tabaci* genome (Guo *et al.* 2015) found that k-mer estimates of one particular biotype were about 60Mb larger than those given by flow cytometry.

An alternative approach to inferring genome size from sequence data are to map NGS reads onto a set of putative single-copy genes using a reference-based assembler to determine the average coverage for the set of genes as a whole, and use that average as an estimate of coverage for the entire genome (Desvillechabrol *et al.* 2016, Kanda *et al.* 2015). Unlike k-mer based estimates, however, this method requires a reference sequence for each locus, and the accuracy of the estimate depends on these loci being truly single-copy.

Despite the potential value of sequence-based genome size estimation methods, little empirical research to verify them has been conducted. In most instances, these methods are incidental to the overall project and are only applied to a single individual or species. In this study, we perform three of these sequence-based genome size estimation techniques, focusing on beetles in the suborder Adephaga, and compare the results to genome size estimates derived from flow cytometry.

## Materials and Methods

### Taxon sampling and specimen processing

Specimens were collected in Oregon and California (Tables S1, S2). Specimens assessed with flow cytometry were collected live, and their heads removed and stored at -80°. The remaining portions of the beetles were stored in 95–100% ethanol and retained as vouchers. Eight of these specimens were also sequenced (Table S2). No flow cytometry was performed on *Amphizoa insolens*, *Omoglymmius hamatus*, and *Trachypachus gibbsii* as sufficient numbers of specimens could not be collected at the time of the study. These three species were only assessed using sequence-based methods.

To insure sufficient single-copy reference sequences would be available for read mapping, six transcriptomes were also sequenced (Table S3). These transcriptomes were derived from whole-body RNA extractions from individual beetles conspecific to those used for genomic sequencing with the exception of *Lionepha casta* DNA4602, which is a close relative of *Lionepha tuulukwa*. Specimens used for transcriptome sequencing were either stored in RNAlater (Thermo Fisher Scientific, Waltham, MA) or kept alive until RNA extraction (Table S4). The reference transcriptome for *Amphizoa insolens* was obtained from the NCBI SRA (accession number: SRR5930489).

### DNA extraction and shallow genome sequencing

DNA was extracted from all specimens using a Qiagen DNeasy Blood and Tissue Kit (Qiagen, Hilden, Germany). DNA was extracted from muscle tissue in most cases; however, in some specimens male reproductive tissue was extracted instead, and in one case (Bembidion lividulum DNA4146) the entire body was extracted (Table S5). The extractions designated *Lionepha tuulukwa* DNA5435 and *Lionepha tuulukwa* DNA5436 were derived from different tissues of the same specimen (reproductive and muscle, respectively) to determine if any obvious qualitative differences resulted from use of different tissue types. Reproductive tissue used included at least one whole testis and accessory gland; the testes of these specimens were large and, given the season of capture, would have been full of cells in various stages of meiosis (primarily pachytene), as well as sperm cells.

The DNA was quantified using a Qubit Fluorometer (Life Technologies, Carlsbad, CA) with a Quant-iT dsDNA HS Assay Kit, and DNA fragment length distributions with a 2100 Bioanalyzer (Agilent Technologies) using the High Sensitivity DNA Analysis Kit. The samples were sheared for 10 min (30 sec on, 30 sec off) to a length of approximately 300bp using a Bioruptor Pico Sonication System (Diagenode, Denville, NJ).

Libraries were prepared using either a NEBNext DNA Ultra II kit (New England BioLabs) or an Illumina TruSeq DNA Sample Prep Kit (Table S5). Illumina sequencing was performed at the Oregon State University Center for Genomic Research and Biocomputing (OSU CGRB) on either a HiSeq 2000 or HiSeq 3000 (Table S5).

### RNA extraction and transcriptome sequencing

All transcriptomic specimens were extracted using Trizol Reagent (Thermo Fisher Scientific) and a Qiagen RNeasy Mini Kit. Whole beetles were homogenized in liquid nitrogen, with the genitalia as well as a single antenna, leg, and elytron being retained as a voucher for each individual. RNA extractions were quantified using a Qubit Fluorometer (Life Technologies) with a Qubit RNA BR Assay Kit (Thermo Fisher Scientific).

The RNA library for *Bembidion* sp. nr. *transversale* DNA3229 was prepared using an Illumina TruSeq RNA Sample Prep Kit at HudsonAlpha Institute for Biotechnology (Huntsville, AL). For all other specimens, mRNA was isolated using NEBNext Poly(A) mRNA Magnetic Isolation Module (New England Biolabs, Ipswich, MA), and libraries were constructed with NEBNext Ultra RNA Library Prep Kit for Illumina (New England Biolabs). The fragment size distribution of each library was characterized with a 2100 Bioanalyzer (Agilent Technologies, Santa Clara, CA) using the High Sensitivity DNA Analysis Kit and 1μl of sample.

*Bembidion* sp. nr. *transversale* DNA3229 was sequenced on an Illumina HiSeq 2000 at the HudsonAlpha Institute for Biotechnology. The remaining transcriptome libraries were run on either an Illumina HiSeq 2000 maintained by the OSU CGRB, or on an Illumina HiSeq 2500 at the Oregon Health and Science University’s Massively Parallel Sequencing Shared Resource (Table S4).

### Read processing and de novo assembly

The reads of one representative of each of the eight species were imported into CLC Genomics Workbench (GW) v9.5.3 (CLC Bio-Qiagen, Aarhus, Denmark), and low quality and Illumina adapter contaminated reads were removed using the “Trim Sequences” tool with a quality limit parameter of 0.05 and an ambiguity limit of 2. *De novo* genome assembly was performed in GW using an automatic word and bubble size. Transcriptome reads were quality and adapter trimmed using the Agalma workflow (Dunn, Howison, and Zapata 2013), and then assembled using Trinity (Grabherr *et al.* 2011). To insure consistent read length for read mapping and k-mer genome size estimation, the raw reads were reprocessed using BBduk v37.62 from the BBTools package (Bushnell 2014). Reads containing Illumina adapters or other NGS artifact sequences, or with an average quality score below 10, were discarded.

The relative quality of assemblies was assessed by identifying single-copy orthologs with BUSCO v3 (Waterhouse *et al.* 2018) using the Endopterygota odb9 reference data set. In order to identify and quantify repetitive elements, a random sample of 500,000 read pairs was generated for each of the eight assemblies and analyzed with RepeatExplorer V2 (Novák *et al.* 2013) with default parameters and using the Metazoa V3 database.

Given that mitochondrial DNA can make up a substantial portion of the DNA present in a cell (Moraes 2001), all libraries were screened for mitochondrial sequence. Mitochondrial DNA sequences were assembled for each genome using NOVOPlasty v2.7.1 (Dierckxsens 2017). NOVOPlasty produced complete circularized mitochondrial genomes for six of the eight libraries (*Amphizoa insolens* DNA3784, *Bembidion lividulum* DNA4161, *Chlaenius sericeus* DNA4821, *Omoglymmius hamatus* DNA3783, *Pterostichus melanarius* DNA3787), while the remaining two libraries (*Bembidion* sp. nr. *transversale* DNA2544 and *Lionepha tuulukwa* DNA3782) produced a single large (>16,000 bp) and several small (<2,500 bp) contigs. The six complete mitochondrial genomes and the two largest contigs from DNA2544 and DNA3782 were combined in a single FASTA file. BBmap was used to map reads from each library to these mitochondrial reference sequences (minid = 0.7), and the unmapped reads were used for subsequent “No Mito” read mapping analyses, which are the primary focus of this paper unless otherwise stated. Although this will also remove from consideration nuclear copies of mitochondrial DNA (“numts”) that are similar enough to the current mitochondrial genome, the fraction of reads that match mtDNA is low enough (0.14–2.81%; see below) that removing numts can have at most a minimal effect on the estimate of genome size, especially as most of the reads that match mtDNA are presumably from the mitochondria.

### Flow cytometry

Genome size was determined following methods in Johnston, Bernardini, and Hjelmen (2019). One half of the head of each frozen adult sample was placed in ice-cold Galbraith buffer, along with the head of a female *Drosophila virilis* strain maintained in the laboratory of JSJ (1C=328 Mb). All specimens of *Bembidion* sp. nr. *transversale* also contained the head of a *Periplaneta americana* (1C = 3,324 Mb) to act as a second internal standard. Combined heads of the sample and standard were ground using 15 strokes of the “A” pestle in a 2 ml Kontes Dounce tissue grinder and filtered through a 40μm nylon mesh. The DNA in the nuclei released by grinding was stained for 2 hr under dark refrigeration with 25 μg/ml propidium iodide. The mean red PI fluorescence of stained nuclei was quantified using a Beckman-Coulter (Brea, CA) CytoFlex flow cytometer with a solid-state laser emitting at 488 nm. The total quantity of DNA in the sample was calculated as the ratio of red fluorescence of sample/standard (mean red fluorescence of the 2C peak of the sample divided by the mean fluorescence of the 2C peak of the standard) times the 1C amount of DNA in the standard. To increase precision, the genome size for each sample was estimated as the average from 2 technical replicates from the two halves of the head of each individual. Up to 5 individuals of each sex were scored to produce biological replicates, allowing calculation of the standard error of the mean genome size estimate. The genome size is reported as 1C, the mean amount of DNA in Mb in one copy of a single complete genome.

### K-mer distribution

The optimal k-mer length for genome size estimation has not been extensively tested, and the choice of k may impact the accuracy of estimates (File S1). At least one study observed that k lengths above 16 resulted in an overestimation of genome size (Panfilio *et al.* 2019), while another observed little variation at different lengths of k (Sun *et al.* 2018). The authors of GenomeScope recommend k = 21 as a good tradeoff between computation speed and accuracy (Vurture *et al.* 2017), though values between 17 and 27 have been used in other studies (Chen *et al.* 2015, Sun *et al.* 2018, Zhang *et al.* 2012). To ensure that the length of k was not affecting the estimates, all k-mer analyses were performed with values of k ranging from 13 to 31, at steps of two, for the four species represented by multiple specimens (*Bembidion* sp. nr. *transversale*, *Chlaenius sericeus*, *Lionepha tuulukwa*, *and Pterostichus melanarius*). One-way ANOVA F-tests were performed for each combination of species and k-mer method (Figures S1–S4).

The input for all k-mer based analyses in this study were plain text histograms depicting the number of k-mers at a given frequency. These were generated using the filtered Illumina reads with Jellyfish v2.0 (Marçais & Kingsford 2011), and genome sizes were estimated using two programs: GenomeScope (Vurture *et al.* 2017) and CovEST (Hozza *et al.* 2015). Histograms were analyzed using GenomeScope with no maximum k-mer coverage (maximum k-mer coverage = -1). GenomeScope uses an analytical model to estimate properties of a genome, including genome size, average coverage, and heterozygosity, from the distribution of k-mer frequencies. The k-mer histograms were also analyzed with CovEST using the “basic” and “repeats” models. CovEST employs a model that accounts for error rate and repeat structure, and has been demonstrated to perform well on low-coverage datasets (Hozza *et al.* 2015).

### Read mapping

Two separate sets of loci were selected for use as reference sequences, both containing putatively single-copy nuclear protein-coding genes. All loci consisted solely of protein-coding, exonic sequence to exclude introns or other non-coding sequence unexpectedly affecting read mapping. The first set contained the 74 single-copy nuclear genes (File S2). This set of loci (here referred to as “Regier”) was initially selected for studying arthropod evolution (Regier *et al.* 2008), and has been used for validating NGS results in previous studies of carabid beetles (Kanda *et al.* 2015, Sproul & Maddison 2017). The reference sequences of these genes for each species were obtained by performing a pairwise search, using Exonerate v2.4.0 (Slater & Birney 2005), of the corresponding transcriptome and the *Bembidion* sp. nr. *transversale* references from Kanda *et al.* (2015). Because no transcriptome was available for *Omoglymmius* or a near relative, reference sequences were generated directly from the assembled *Omoglymmius hamatus* DNA3783 genome.

Read mapping was repeated with a second non-overlapping set of putatively single-copy genes from OrthoDB v9.1 (Zdobnov *et al.* 2016); this set is here referred to as “ODB” (File S3). Loci from six coleopteran genomes were obtained (*Agrilus planipennis*, GCF_000699045.1; *Anoplophora glabripennis*, GCF_000390285.2; *Dendroctonus ponderosae*, GCF_000355655.1; *Leptinotarsa decemlineata*, GCF_000500325.1; *Onthophagus taurus*, GCF_000648695.1; *Tribolium castaneum*, GCF_000002335.3) by filtering for genes present in >80% of species at the Endopterygota level, resulting in a total of 135 loci (File S4). Next, the loci were used as reference sequences for an orthologous gene search of the eight adephagan transcriptomes created here using the Orthograph software package (Petersen *et al.* 2017). Orthograph uses profile hidden Markov models derived from the amino acid sequences of predetermined ortholog groups from related species to identify orthologous genes in novel NGS data. These putative orthologs are then validated by a reciprocal BLAST using the same set of orthologous gene groups.

**Figure 1:**
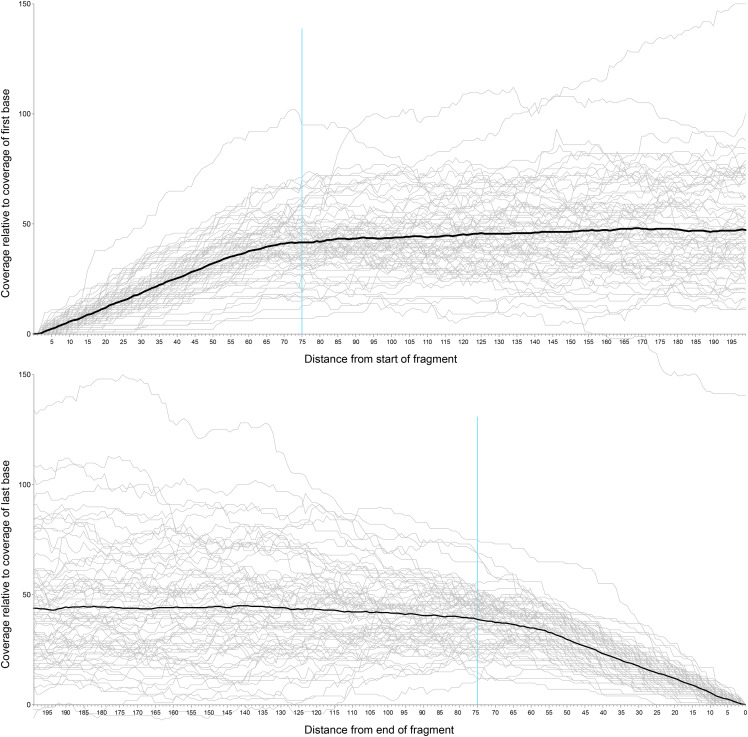
Read mapping coverage at the beginning and end of each of the Regier set loci using reads from *Bembidion* sp. nr. *transversale* DNA2544. The black line indicates the average relative coverage along the length of the locus, and the blue line shows 75 base positions from either end of the locus.

Read mapping for both data sets was performed using BBmap v37.62 with the minid parameter set to 0.7. Preprocessed reads from each of the genomic libraries were mapped to the each of the gene sets. Sequencing coverage for each library was estimated by calculating the mean coverage across all loci. The raw coverage data were generated using BBmap’s per-scaffold coverage output (using the *basecov* option). We generated plots showing coverage at each nucleotide position of the reference loci (Figures 1, S5) and observed that the coverage profile varied across each locus, with the first and last 60 to 70 bases having substantially lower coverage than the central region of the locus. Some variation in coverage is inevitable due to the stochastic nature of shotgun sequencing (Lindner *et al.* 2013, Desvillechabrol *et al.* 2016); however, the decreased coverage toward the flanks is likely an artifact of the read mapping procedure, since BBMap does not map a read if only a small portion of the read overlaps with the reference sequence.

To eliminate bias from small read overlaps with reference sequences, a custom python script, GSEC (https://github.com/JMPflug/gsec), was made to trim low coverage, flanking regions of the read mapping and produce corrected coverage estimates. The script begins with one “per-base” coverage file, containing the coverage at each base position of all loci in the reference set, for each sequenced library. This type of file can be generated by several read mapping utilities, including BBMap, Samtools, and Bedtools. The script then excludes a user-selected number of base positions at the beginning and end of each locus. The coverage profiles of the Regier set mappings for several specimens (Figures 1, S5) indicated that the drop-off in relative coverage begins at roughly 75 bases from the ends of each locus; we chose this value as the number of bases excluded by the script from each end. To prevent outlier loci from skewing the calculated means, we included loci with coverage and length values within three interquartile ranges of the median; loci with more extreme coverage or length values were removed. The script then recalculates the average per-base coverage of each locus in the reference set and uses these values to calculate the average genome coverage for the library across all loci. These average coverage values are then used as an estimate of the average coverage of the genome, and genome size is calculated using the Lander-Waterman equation (Lander and Waterman 1988),

$$G=\frac{LN}{C}$$

where C is the coverage estimated by read mapping, L is read length, N is the total number of reads, and G is the haploid genome length.

### Model species

We repeated the previously described k-mer and read mapping methods using Illumina reads from the NCBI Short Read Archive (SRA) from three model organism species, *Arabidopsis thaliana*, *Caenorhabditis elegans*, and *Drosophila melanogaster*, with each species being represented by four separate libraries (Table S14). All four *D. melanogaster* specimens were female. For read mapping estimates, we used the latest available genome assembly from GenBank: *A. thaliana*, GCA_902460285.1 (Berardini *et al.* 2015); *C. elegans*, GCA_000002985.3 (Harris *et al.* 2020); and *D. melanogaster*, GCF_000001215.4 (Thurmond *et al.* 2018). BUSCO was used to produce a set of single copy orthologs for each model species to serve as references. We used the embryophyta_odb10, nematoda_odb9, and diptera_odb9 BUSCO databases for *A. thaliana*, *C. elegans*, and *D. melanogaster*, respectively.

### Karyotyping

Although not essential for determining genome size, karyotyping can provide evidence for major structural changes to genomes, such as chromosome duplication or loss, or whole genome duplication. Two species examined in this study, *Bembidion* sp. nr. *transversale* and *Lionepha tuulukwa*, lacked published chromosome counts. A total of 14 males of *Bembidion* sp. nr. *transversale* and three males of *Lionepha tuulukwa* were examined for chromosome number and size, with specimens collected from the same localities studied for genome size. In brief, Feulgen staining followed by squashing was used; more details are given in Maddison (1985). First and second meiotic metaphase and anaphase were studied to determine chromosome number and sex chromosome system.

### Data availability

Raw genomic reads were deposited in the Short Read Archive of NCBI’s GenBank database under accession numbers SRR8518612 to SRR8518632 (Table 1), and transcriptome reads were deposited to the NCBI SRA under accession numbers SRR8801541 to SRR8801545 (Table S3). The custom python script used is available at: https://github.com/JMPflug/gsec. Supplemental material available at figshare: https://doi.org/10.25387/g3.12497855.

**Table 1:** Average flow cytometry genome size measurements. Values given in Mb. SD indicates standard deviation; SE indicates standard error

	Female				Male			
	Genome Size	N	SD	SE	Genome Size	N	SD	SE
*Bembidion sp. nr. transversale*	2193.41	9	26.26	8.75	2118.05	9	27.77	9.26
*Bembidion lividulum*	837.39	1	—	—	831.70	5	6.57	2.94
*Chlaenius sericeus*	408.37	6	12.43	5.08	391.50	4	4.01	2.00
*Lionepha tuulukwa*	597.60	1	—	—	585.70	5	5.41	2.42
*Pterostichus melanarius*	1040.65	4	18.42	9.21	1000.93	4	19.16	9.58

## Results

### Flow cytometry

**Figure 2:**
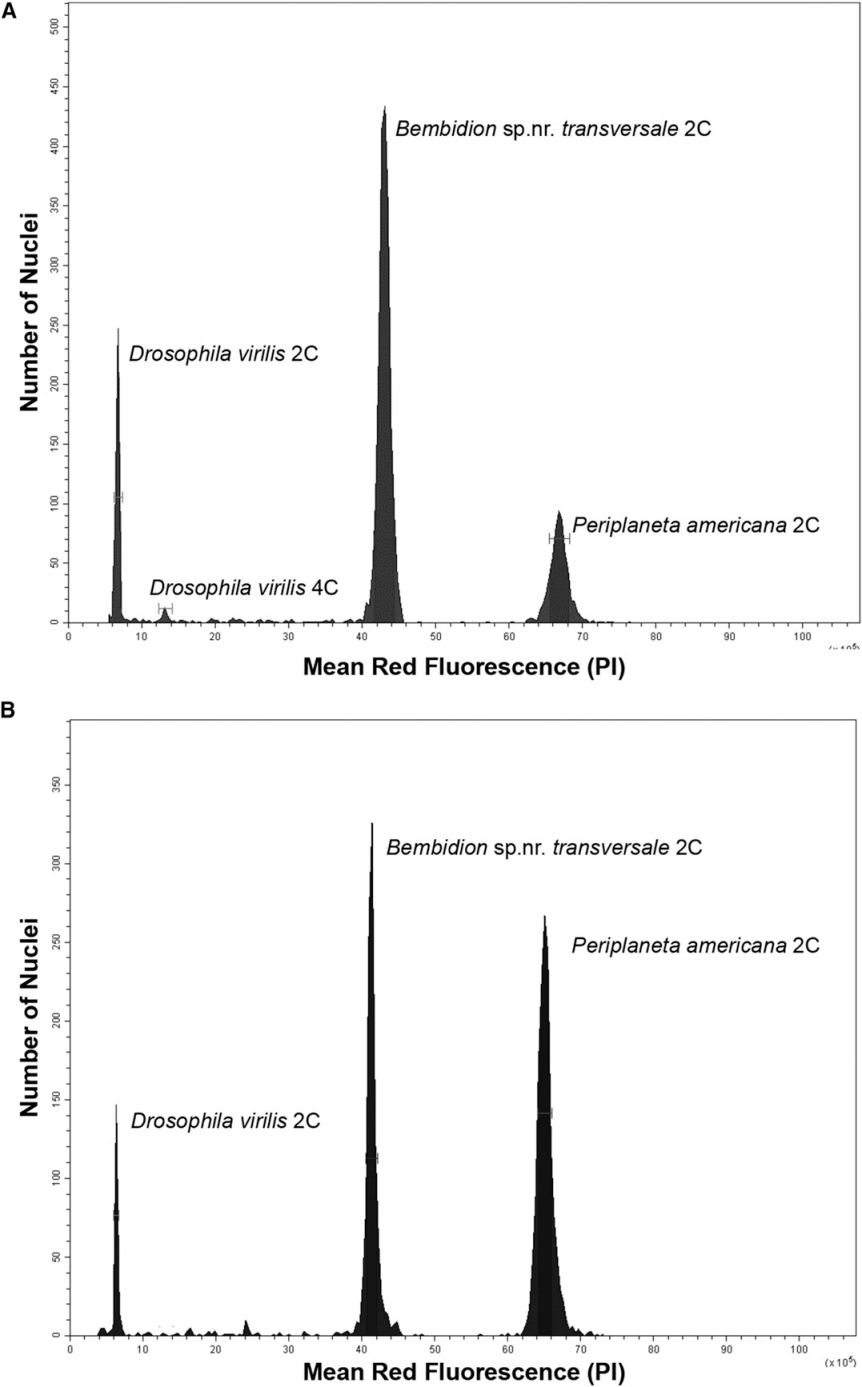
Relative red fluorescence and the number of nuclei counted at each fluorescence level of representative male (A) and female (B) *Bembidion sp. nr. transversale*. Bars around each peak represent statistical gates that provide the total nuclei in that peak, average channel number of nuclei in the peak, and the coefficient of variation (CV). *D. virilis* standard 1C = 328 Mb, *P. americana* standard 1C = 3,338 Mb.

The genome sizes of the five species of carabid beetles studied (Tables 1, S2) vary over a fivefold range. *Bembidion* sp. nr. *transversale* possessed the largest genome, with estimates of 1C = 2,193.4♂ / 2,118.1♀Mb (Figure 2), while the genome of *Chlaenius sericeus* was the smallest with 1C = 408.4♂ / 391.5♀Mb (Figure S6). The genome of the former is over twice the size of the largest previously measured carabid beetle, *Calosoma scrutator*, which was estimated to be 1C = 1,017.1Mb (Hanrahan and Johnston 2011), and ranks as the 8^th^ largest beetle genome out of the nearly 300 analyzed Coleoptera in the Animal Genome Size Database (Gregory 2020).

### Sequencing and assembly

Approximately 3.5 billion reads were generated across all 21 genomes, resulting in a total 473.6 billion bases. These were used to create draft assemblies of the eight species of adephagan beetles studied. The total assembled scaffold lengths varied between 153.1Mb for *Omoglymmius hamatus* DNA3783, and 665.1Mb for *Bembidion* sp. nr. *transversale* DNA2544 (Table 2). The assembly lengths are substantially smaller than the genome sizes inferred by both sequence-based and flow cytometric methods, indicating that large portions of the genomes could not be assembled. The number of genes in each assembly identified by BUSCO as “complete” (*i.e.*, a putative orthologous gene found similar to one of the 2442 BUSCO gene groups, and whose length is within two standard deviations of the mean length of the genes in that BUSCO group) varied greatly across the genome assemblies (Table S6). 1,923 (78.7%) of the 2442 genes were completely found in *Chlaenius sericeus* DNA4821, while *Omoglymmius hamatus* DNA3783 contained 178 (7.3%) complete genes.

**Table 2:** Summary of results for genomes assembled with CLC Genomics Workbench

	Read Length	Total Reads	Reads After Trim	N50	Average Contig (bp)	Maximum Contig (bp)	Contig Count	Total Assembled Bases
*Amphizoa insolens* DNA3784	101	59,590,984	59,182,421	355	299	79,486	905,365	270,465,134
*Bembidion sp. nr. transversale* DNA2544	151	717,036,094	713,035,405	525	460	140,578	1,438,922	661,385,315
*Bembidion lividulum* DNA4161	101	55,816,204	51,442,737	673	543	16,894	280,998	152,578,104
*Chlaenius sericeus* DNA4821	151	77,809,004	75,284,196	1,411	893	68,744	340,400	304,113,552
*Lionepha tuulukwa* DNA3782	101	80,214,318	79,962,073	4,114	1,481	47,683	124,458	184,268,766
*Omoglymmius hamatus* DNA3783	101	80,209,476	79,995,111	366	306	13,857	1,298,649	397,294,330
*Pterostichus melanarius* DNA3787	101	69,486,602	69,476,201	341	289	17,107	1,160,880	335,949,421
*Trachypachus gibbsii* DNA3786	101	88,000,000	86,431,416	1,258	588	65,825	472,429	277,787,110

A total of over 570 million reads were generated from the six transcriptomes (Table 3). The number of transcripts assembled ranged from 22,330 with *Bembidion lividulum* DNA4279 to 57,119 with *Bembidion* sp. nr. *transversale* DNA3229. As anticipated, BUSCO was able to locate many more genes in the transcriptomic assemblies (Table S7).

RepeatExplorer classified between 23.9% and 66.6% of the sampled reads as originating from repetitive elements (Figure S7). Three samples, all from the carabid tribe Bembidiini, consisted of over 50% repetitive DNA: *Bembidion* sp. nr. *transversale* DNA2544, *Lionepha tuulukwa* DNA3782, and *Bembidion lividulum* DNA4161. RepeatExplorer identified a variety of repeat families, including Ty3/Gypsy, Ty1/Copia, Penelope, and LINEs; however, most repetitive sequences were placed in the general “unclassified” category (70.1–99.3%). 10.3% of the *Bembidion lividulum* DNA4161 reads were classified as ribosomal DNA, a finding consistent with other studies on this species (Sproul and Maddison 2017). The genomic libraries consisted of between 0.14–2.81% mitochondrial DNA.

**Table 3:** Summary of results for transcriptomes assembled with Trinity

	Read Length	Reads Examined	Transcripts	N50	Average contig length	Total assembled bases
*Bembidion sp. nr. transversale* DNA3229	50	425,577,514	57,119	1274	898.66	35,767,428
*Bembidion lividulum* DNA4279	101	22,400,532	22,330	1727	1177.05	20,375,906
*Chlaenius sericeus* JMPR007	101	22,103,790	24,327	1764	1146.54	22,566,122
*Lionepha casta* DNA4602	100	30,791,800	26,994	1963	1338.76	23,724,243
*Pterostichus melanarius* DNA4765	100	38,061,712	34,153	2184	1338.91	30,509,665
*Trachypachus gibbsii* DNA4436	101	31,571,972	29,159	1823	1209.21	26,260,335

### K-mer distribution

The estimated genome size changed with k-mer length for both CovEST models, but not for GenomeScope (Table 4). The length of k had a significant effect on estimated genome size in all four species (one-way ANOVA, p-value < 0.001) for both “repeats” and “basic” models. A non-significant difference in GenomeScope genome size estimates was observed at varying sizes of k for all species (one-way ANOVA, p-value > 0.40).

**Table 4:** Summary of genome size estimates using flow cytometry and sequence-based methods. Values given in Mb. Flow cytometry Flow cytometry was not performed on *Trachypachus gibbsii* DNA3786, *Amphizoa insolens* DNA3784, and *Omoglymmius hamatus* DNA3782. CovEST Basic, CovEST Repeat, and GenomeScope analyses were conducted using a k value of 21. Cells in the GenomeScope column containing dashes indicate the sample failed to converge

	Flow Cytometry	Regier Mapping	ODB Mapping	GenomeScope	CovEST Basic	CovEST Repeat
*Amphizoa insolens* DNA3784	—	710.1	610.7	—	376.78	728.47
*Bembidion sp. nr. transversale* DNA2544	2,118.1*^a^*	1,291.1	1,241.0	1,113.67	932.04	2,140.04
*Bembidion sp. nr. transversale* DNA5427	2,118.1*^a^*	1,114.9	1,113.4	1,010.67	827.88	1,980.24
*Bembidion sp. nr. transversale* DNA5428	2,118.1*^a^*	897.9	866.4	945.51	813.44	1,924.27
*Bembidion sp. nr. transversale* DNA5433	2,118.1*^a^*	983.3	946.4	908.41	758.09	1,480.15
*Bembidion lividulum* DNA4161	831.7*^a^*	603.1	645.8	—	359.94	790.81
*Chlaenius sericeus* DNA4821	391.5	411.5	438.5	414.46	386.92	608.44
*Chlaenius sericeus* JMP068	385.8	390.1	395.7	374.46	410.91	751.89
*Chlaenius sericeus* JMP069	390.1	389.5	393.4	374.67	395.86	704.51
*Chlaenius sericeus* JMP070	396.5	354.5	393.1	—	409.81	796.39
*Chlaenius sericeus* JMP071	393.6	383.1	406.7	377.09	412.78	624.53
*Lionepha tuulukwa* DNA3782	585.7*^a^*	769.1	663.1	545.72	442.32	659.97
*Lionepha tuulukwa* DNA5435*^b^*	585.7*^a^*	518.1	535.9	483.39	340.38	423.66
*Lionepha tuulukwa* DNA5436*^b^*	585.7*^a^*	522.1	513.9	493.63	346.52	578.96
*Omoglymmius hamatus* DNA3783	—	1,525.7	1,552.1	74.03	627.13	1,188.94
*Pterostichus melanarius* DNA3787	1,000.9	1,144.9	984.6	74.58	475.11	1,221.83
*Pterostichus melanarius* JMP059	1,045.1	760.3	860.5	—	586.67	1,127.86
*Pterostichus melanarius* JMP060	1,018.3	855.5	787.4	—	597.29	1,145.04
*Pterostichus melanarius* JMP061	1,068.0	859.9	746.4	—	650.22	1,454.54
*Pterostichus melanarius* JMP062	1,031.2	1,103.7	927.6	—	678.61	1,181.38
*Trachypachus gibbsii* DNA3786	—	342.0	315.5	264.08	314.81	525.13

a Flow cytometry measurements for sample are species averages of multiple individuals (see Tables 2 and S3).

b Samples made with DNA extracted from different tissues of the same individual.

Post-hoc Tukey-Kramer tests of the CovEST results revealed that estimates using a k of less than 17 differed significantly (p-value < 0.05) from those using k values above 21 for all species (Figures S1–S4). Values of k less than 17 always yielded much lower genome size estimates than suggested by both flow cytometry and read mapping, while larger k values (19 to 31) produced estimates that were more consistent with the results of other estimation methods. Given these observations, and as the computational expense of generating k-mer graphs increases as k becomes larger (Marçais & Kingsford 2011), a k value of 21 was selected as the standard k value for this study. All subsequent discussion about k-mer based estimates in this study, including tables and figures, refer to analyses performed using this value unless otherwise stated.

GenomeScope converged and produced genome size estimates for 14 of the 21 specimens (Table S8). GenomeScope failed to converge for *Amphizoa insolens* DNA3784, *Bembidion lividulum* DNA4161, *Chlaenius sericeus* JMP070, and four of the five *Pterostichus melanarius* samples (JMP059, JMP060, JMP061, and JMP062). *Omoglymmius hamatus* DNA3783 and *Pterostichus melanarius* DNA3787 converged but yielded implausibly low genome size estimates (74Mb and 74.6MB, respectively). The specimens that failed to converge lacked obvious coverage peaks in their k-mer histograms, suggesting they did not have sufficient coverage for the GenomeScope model. Coverage estimates of these specimens using read mapping provide support for this idea, as all specimens that did not converge had an estimated coverage less than 26X, while only one specimen whose coverage was less than 26X, *Lionepha tuulukwa* DNA3782, converged (Tables S9, S10). A similar situation was observed for both CovEST models. All non-converging specimens were estimated to have a coverage less than 13X for the “basic” model and less than 22X for the “repeats” model, with *Lionepha tuulukwa* DNA3782 again being the only specimen to converge with a coverage below this level. This result is consistent with the Vurture *et al.* (2017) recommended minimum coverage of at least 25X coverage for an accurate estimate. The successful analyses produced genome size estimates ranging from 1,113.7Mb for *Bembidion* sp. nr. *transversale* DNA2544 to 264.0Mb for *Trachypachus gibbsii* DNA3786.

CovEST produced estimates for all 21 samples (Tables S6, S10), though the two models behaved differently. The “repeats” model yielded estimates approximately double the size of the “basic” model estimates, and the “repeats” model always produced the largest estimate among all the sequence-based methods.

### Read mapping

There was no statistically significant difference between the estimates using the Regier and ODB gene sets (two-sample *t*-test, *P* = 0.1611). Removing mitochondrial reads resulted, on average, in a modest increase in genome size estimates for both Regier (1.31%, s = 0.75%) and ODB (1.2%, s = 0.66%) gene sets (Table S9).

**Figure 3:**
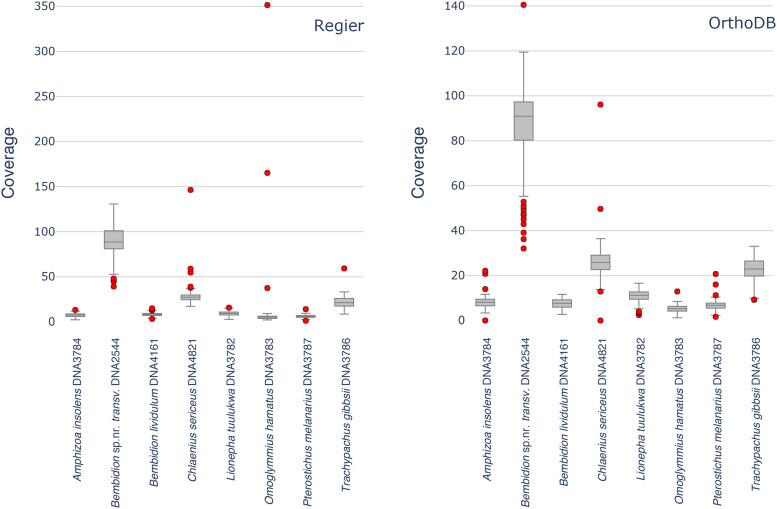
Boxplot of average genomic read mapping coverages for each of the Regier (left) and OrthoDB (right) genes for eight representative specimens. Red dots indicate outlier genes with coverage outside three interquartiles from the median.

In most samples, we observed that a small number of loci with notably high coverage were removed by the GSEC script. In all but one instance, excluding these loci had a minimal impact on the average coverage (Tables S11, S12). The single exception, *Omoglymmius hamatus* DNA3783, contained three Regier set loci with substantially higher coverage (>160X) than the rest of the set (Figure 3), which ranged between 2.10X to 9.27X. The inclusion of these three loci nearly doubled the estimated genome size. The high-coverage of these loci suggests that all or part of these genes are not single copy in this species, and that their apparent coverage is being inflated by copy number variation or extraneous reads from an undetected source such as a pseudogene or contaminant DNA from another organism.

### Comparison of flow cytometry and sequence-based genome size estimates

**Figure 4:**
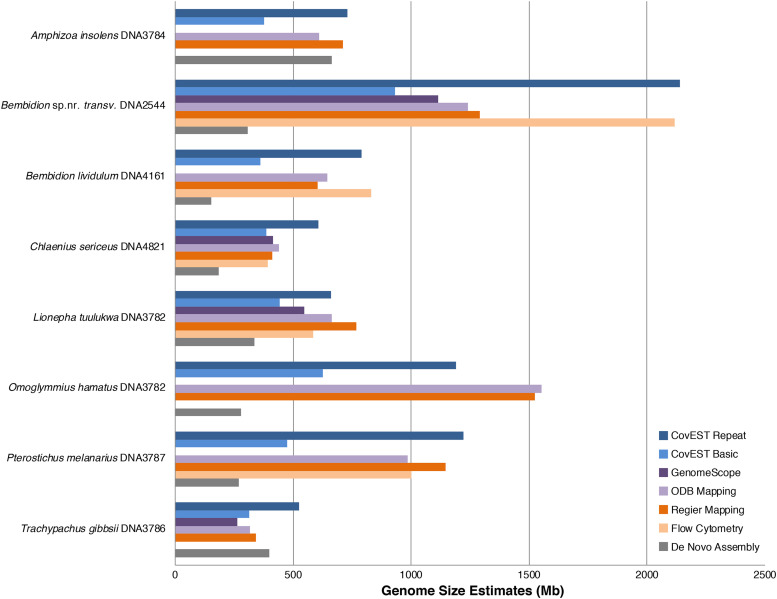
Summary of genome size estimates using flow cytometry and sequence-based methods for the eight adephagan species. Flow cytometry measurements are averages of multiple individuals (see Tables 2 and S3). CovEST Basic, CovEST Repeat, and GenomeScope analyses were conducted using a k value of 21. Sequence-based estimates were obtained from different individual specimens (Table 1) than those analyzed with flow cytometry.

**Figure 5:**
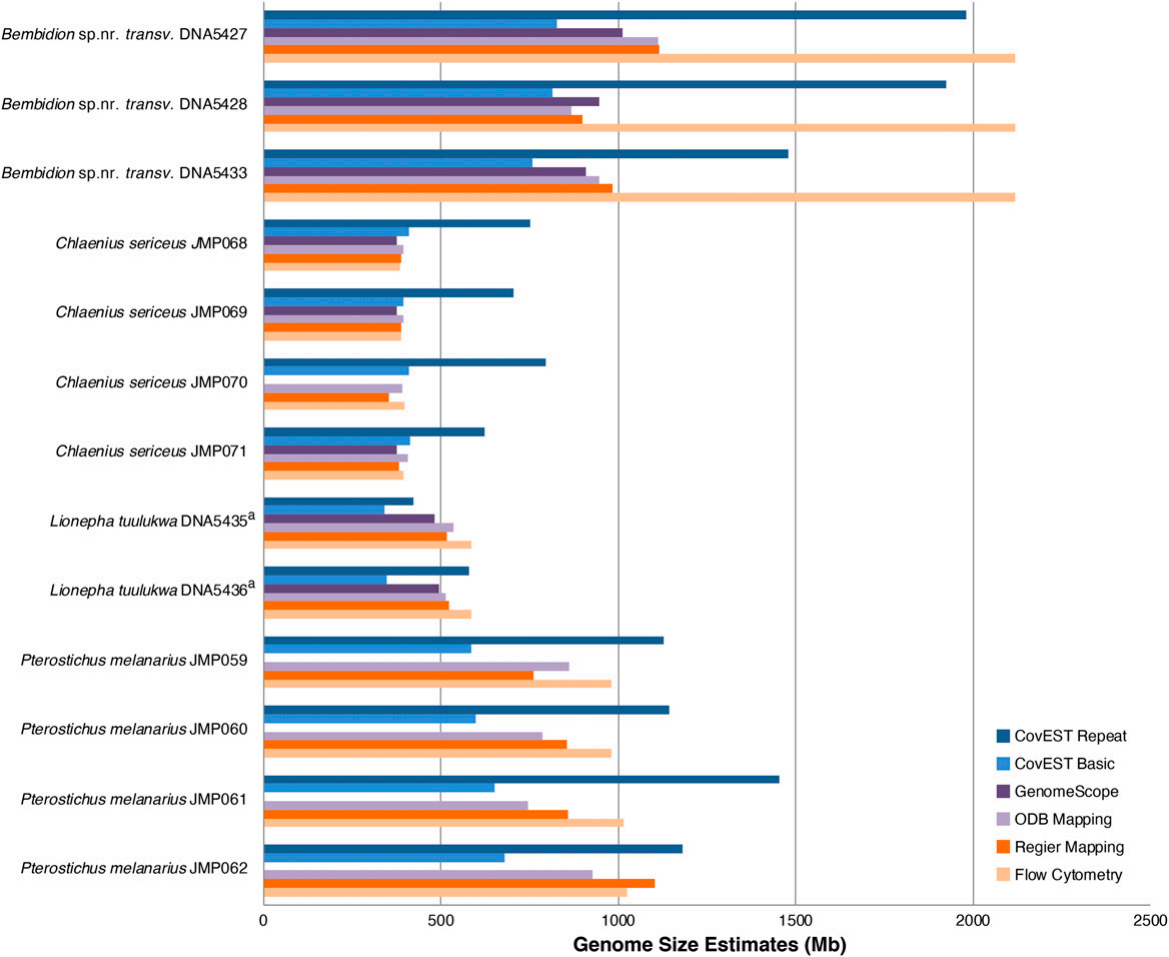
Summary of genome size estimates using flow cytometry and sequence-based methods for the 13 samples. CovEST Basic, CovEST Repeat, and GenomeScope analyses were conducted using a k value of 21. a. Samples made with DNA extracted from different tissues of the same individual.

The accuracy of sequence-based genome size estimation methods depended greatly on the species being analyzed (Figures 4, 5). Sequence-based methods underestimated size of the genome as measured by flow cytometry by an average of 11%. This general pattern has also been observed when comparing whole genome assemblies to flow cytometry and Feulgen staining (Elliott and Gregory 2015). In addition, estimates produced by the various sequence-based methods sometimes differed from each other. The largest discrepancy among methods was observed with the large genome of *Bembidion* sp. nr. *transversale*. The average flow cytometry measurement of male *Bembidion* sp. nr. *transversale* was 2,118.1Mb; however, read mapping, GenomeScope, and the “basic” CovEST model estimated the genome to be approximately half that size (932.0Mb to 1134.1Mb). A similar, though less pronounced, pattern was observed with the next largest genome, of *Pterostichus melanarius*. In contrast, the CovEST “repeats” estimate was within 10% of the flow cytometric value in three of the four *Bembidion* sp. nr. *transversale* samples (Table S6). For *Chlaenius sericeus*, read mapping, GenomeScope, and CovEST “basic” underestimated genome size by a modest 4.50% on average, while the CovEST “repeats” model produced the only overestimate, inflating the genome by an average of 78.0%. Sequence-based estimates of *Lionepha tuulukwa* varied depending on the method but were generally lower than the flow cytometric value.

### Model species

Overall, the sequence-based estimates of the three model species were usually within 25% of the reported genome size (Table S14), ranging from an overestimate of 22% to an underestimate of 13.5%. Across the three species, the average estimate for the CovEST “repeats” model was closest to the expected value (Table S15), deviating from the reported genome size by an average of 7%, followed by read mapping (10.6%) and GenomeScope (13.3%). The CovEST “basic” model performed the worst, differing by an average of 17.9%.

### Karyotype results

**Figure 6:**
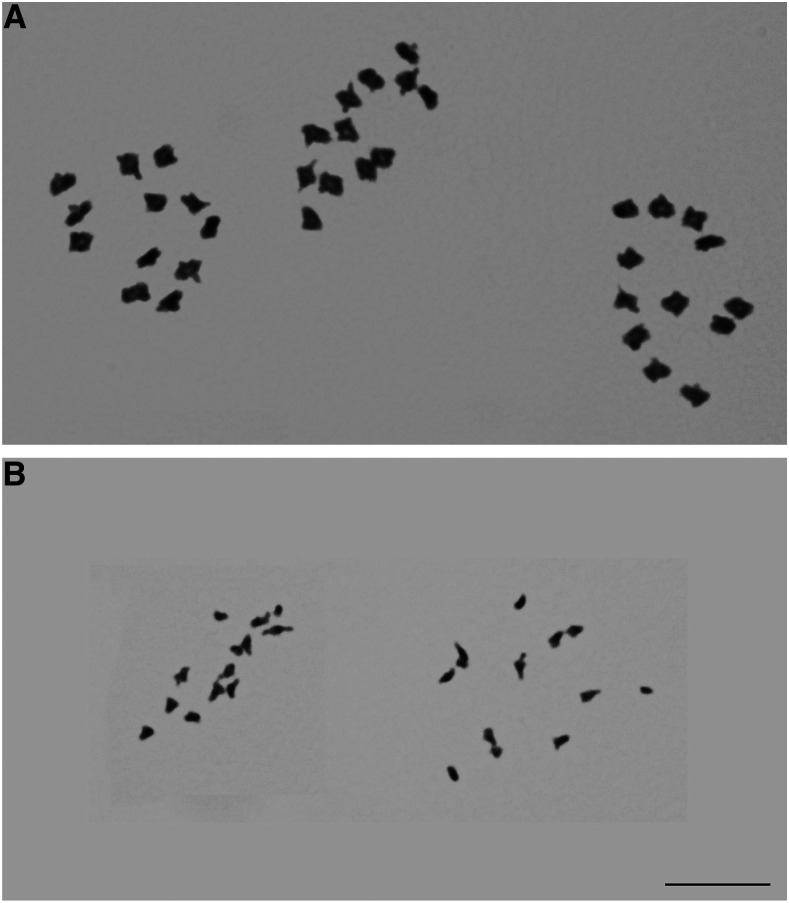
Meiotic first metaphase cells. (A) Three cells of male *Bembidion sp. nr. transversale* (B) Two cells of male *Lionepha tuulukwa*. Photographs are at the same scale. Scale bar 10µm.

Males of *Bembidion* sp. nr. *transversale* have 11 pairs of autosomes and an XY pair of sex chromosomes; males of *Lionepha tuulukwa* have 12 pairs of autosomes and have a single X chromosome with no Y chromosome (Figure 6). These chromosome counts are the same as the near relatives of both species (Maddison 1985, Maddison and Sproul 2020), which suggests no large-scale changes in chromosome number have occurred in their recent evolutionary history.

## Discussion

### K-mer analysis

The genome size estimates from k-mer methods were generally similar to flow cytometry estimates for most species, but the discrepancy seen with *Bembidion* sp. nr. *transversale* shows that these methods can be unpredictable in some cases. With the exception of CovEST “repeats”, the sequence-based estimates for all four *Bembidion* sp. nr. *transversale* specimens were around half the size suggested by the flow cytometry measurements. Closer inspection of the GenomeScope results showed that the estimated coverage was approximately double the value expected for a species with a 2,118Mb haploid genome, leading to the underestimated genome size. The k-mer histograms of *Bembidion* sp. nr. *transversale* DNA2544, the specimen with by far the most reads, showed a distinct bimodal profile, with a large peak at 40X coverage and a shorter peak at 80X. This is potentially indicative of a highly heterozygous genome (Vurture *et al.* 2017). Additionally, GenomeScope estimated that all four *Bembidion* sp. nr. *transversale* genomes consisted of upwards of 75% repetitive sequence, which is similar to the RepeatExplorer estimate (Table S13). A previous study has found that repeats, as well as high heterozygosity and sequencing errors, can decrease the accuracy of genome size estimation using k-mer frequency (Liu *et al.* 2013). This suggests the large number of repetitive sequences in the *Bembidion* sp. nr. *transversale* genome may be at least partially responsible for the observed underestimates.

The striking difference between the estimates of the two CovEST models, with “repeats” consistently giving estimates twice the size of “basic,” suggests that neither model is well suited to all genomes. Genome size estimates of the five *Chlaenius sericeus* specimens were closer to the true value using the “basic” model, while the opposite was true of the five *Pterostichus melanarius* specimens. The CovEST “repeats” model was the sequence-based method which came closest to correctly estimating the 2,100Mb genome of *Bembidion* sp. nr. *transversale* with estimates ranging between 1,480Mb to 2,140Mb. A similar pattern was observed in the CovEST estimates for the three model organisms (Table S15), with the CovEST “repeats” model estimate being consistently higher than the “basic” model. GenomeScope estimates were very close to the expected 100Mb *C. elegans* genome, but were between 25 and 40Mb smaller than the expected values for *A. thaliana* and *D. melanogaster*.

### Read mapping

The method we used to infer coverage from read mapping data (averaging the coverage across many single-copy loci) is relatively simple. Despite its simplicity, this approach managed to perform well in *Chlaenius sericeus* and *Lionepha tuulukwa*, which had smaller genomes. Read mapping proved to be inconsistent when estimating genome size in *Pterostichus melanarius*, and as with GenomeScope, it consistently underestimated *Bembidion* sp. nr. *transversale* by approximately half. For the three model organisms, read mapping performed, on average, slightly better than GenomeScope, and slightly worse than the CovEST “repeats” model.

The reason for the underestimation of *Bembidion* sp. nr. *transversale* genome size based on single-copy read coverage is unclear. Unlike k-mer based methods, which can struggle to assess highly repetitive genomes, the read mapping approach used in this study only infers coverage from single-copy exonic regions. In principle, as long as the selected loci are truly single-copy and the resulting sequence data exhibit no biases in regions sequenced, the estimated coverage should approach the true coverage. It follows that the presence of repetitive sequences elsewhere in the genome should have no effect on this estimate. In actual genomes, evolutionary processes responsible for increasing the size of the genome, such as segmental duplication followed by neofunctionalization or pseudogenization, can lead to the proliferation of genes with similar sequences (Rastogi and Liberles 2005, Levasseur and Pontarotti 2011). Such processes can complicate the selection of a single-copy reference gene set, especially if the group of organisms being studied lacks rich genomic resources; however, these processes are expected to be localized to specific regions of the genome. Barring a large-scale change such as the duplication of the whole genome (a possibility we discuss in the next section), a large sampling of loci and the removal of outliers should compensate for the presence of a small number of duplicate loci.

We considered the possibility that using a larger set of reference loci from *Bembidion* sp. nr. *transversale* may yield a more accurate coverage estimate. To test this, we repeated read mapping on *Bembidion* sp. nr. *transversale* DNA2544 using the 1421 single-copy orthologs annotated by BUSCO and “No Mito” reads. However, this gave coverage and genome size estimates (94.89X and 1,115.9Mb, respectively) that were very similar to the Regier (96.18X, 1,101Mb) and ODB (93.36X, 1,134Mb) gene sets, which casts doubt on insufficient references as the cause.

### Comparison of flow cytometry and sequence-based genome size estimates

No single sequence-based estimation method proved to be accurate in all cases. Species with large genomes, such as *Bembidion* sp. nr. *transversale* and *Pterostichus melanarius*, appear to present the greatest difficulty for inference of genome size by sequence-based means alone. It is possible that the same factors responsible for inaccuracy of k-mer methods are at work in read mapping, especially given that the two methods often yielded underestimates of similar magnitude. However, this study does not provide clear evidence as to what exactly those factors may be. Although both methods use the same underlying reads to estimate coverage, they rely on somewhat different assumptions and portions of the genome. For example, read mapping focuses on coding regions, but k-mer analysis analyzes entire genomes (Vurture *et al.* 2017).

The underestimation of the *Bembidion* sp. nr. *transversale* genome by half by most sequence-based methods, including read mapping, is particularly puzzling. A potential explanation that could have accounted for this is recent whole genome duplication or copy number variation. Evidence of whole genome duplication events is lacking in most groups of insects (Roelofs *et al.* 2020), and no such events have been previously documented in *Bembidion* or near relatives (Maddison 1985, Serrano 1981, Serrano and Galian 1998). The chromosome counts we obtained from karyotyping male reproductive tissue, 11 pairs of autosomes and an XY pair of sex chromosomes (2n = 22+XY), is identical to all known near relatives of *Bembidion* sp. nr. *transversale*, and is the ancestral state throughout most of the multimillion year history of the large genus *Bembidion* (Maddison 1985); this suggests no such duplication event has occurred in the ancestors of *Bembidion* sp. nr. *transversale* within the recent evolutionary past. Analysis of *Bembidion* sp. nr. *transversale* DNA2544 sequence data using the program Smudgeplot (Ranallo-Benavidez *et al.* 2020) also failed to provide evidence of polyploidy. This program, which uses the ratio of heterozygous k-mer pairs to estimate ploidy, indicated that *Bembidion* sp. nr. *transversale* is likely diploid (Figure S8).

Flow cytometry also showed no evidence of genome duplication or endopolyploidy in *Bembidion* sp. nr. *transversale*. One of the advantages of flow cytometry is that it scores genome size in thousands of nuclei and will produce cleanly separate peaks for each ploidy level (Galbraith *et al.* 1991). While acquired somatic diploidy is known for haploid males in Hymenoptera (Aron *et al.* 2005), and while polyteny and endpolyploidy is common in insect tissues, there are no known examples where the basal genome size, whether haploid or euploid, is replaced entirely. Even with acquired somatic diploidy, endopolyploidy and polyteny, large numbers of nuclei remain unreplicated at G_0_ and form a tight peak that is easily recognized and scored to estimate genome size (Johnston, Bernardini & Hjelmen 2019). The flow cytometry histograms observed from *Bembidion* sp. nr. *transversale* tissue (Figure 2) are typical of diploid cells, showing a single clean 2C peak, with no indication of additional peaks suggesting large numbers of cells at higher ploidy levels.

Although no evidence of polyploidy was detected, *Bembidion* chromosomes do possess several unusual characteristics. The number and shape of chromosomes is remarkably consistent among species, with almost all species having males with 2n = 22+XY, a value shared by nearly all *Bembidion*, including *Bembidion* sp. nr. *transversale* (Maddison 1985). Each autosome consists primarily of a large heterochromatic central region flanked by small euchromatic tails, and males exhibit achiasmatic meiosis (Maddison 1985, Serrano 1981, Serrano and Galian 1998). Several species of *Bembidion*, including *Bembidion lividulum*, are also known to possess highly replicated rDNA regions (Sproul and Maddison 2017). It is possible that some of these chromosomal properties are involved in the underestimation of genome size by sequence-based methods.

## Conclusions

Increasing our knowledge of genome sizes across the tree of life is important for a deeper understanding of genomic evolution, but the pace of this increase is currently very slow. In this paper, we have presented the first published flow cytometry estimates for five species of carabid beetles. Although this nearly doubles the number of carabids with genome size estimates, we now have flow-cytometric estimates for less than 0.03% of carabid species.

With the explosion of short-read sequence data, the development of methods to infer genome sizes from such bioinformatic data could markedly increase the pace at which new genome sizes are measured. Unfortunately, these methods presented several problems that may limit their usefulness. While some sequence-based methods were consistent with flow cytometry in some species, we found that no single technique was uniformly congruent with flow cytometry. Flow cytometry or Feulgen densitometry should be the preferred option for estimating genome size when live material and adequate resources are available. In cases where this is not possible, especially when working with rare or extinct organisms, sequence-based methods can provide an initial estimate of the size of a genome. These methods may also be well suited for cases when the genome is likely to be small and non-repetitive. However, our work shows that these techniques can be misleading, particularly when read coverage is low, so researchers should be mindful of the uncertainty.

As of now, sequence-based genome size estimates are best thought of as rough, provisional approximations of genome size, rather than a replacement for conventional cytometric methods. As the cost of NGS continues to decrease and sequencing of novel, non-model species becomes more ubiquitous, these techniques will likely become even more popular tools in the bioinformatics toolbox, so understanding their potential shortcomings is all the more important.
